# Borderline personality disorder: a spurious condition unsupported by science that should be abandoned

**DOI:** 10.1177/01410768231164780

**Published:** 2023-04-12

**Authors:** Roger Mulder, Peter Tyrer

**Affiliations:** 1 Department of Psychological Medicine, University of Otago, Christchurch, 4 Oxford Terrace, Christchurch 8011, New Zealand; 2 Imperial College, Hammersmith Hospital, London, W12 0NN, UK

Twenty years ago, George Vaillant, in a paper entitled ‘The Beginning of Wisdom is Never Calling a Patient a Borderline’^1^ noted that the diagnosis of borderline often reflects the clinician’s affective state rather than careful assessment. This was not an isolated opinion, but we argue that little has changed and that borderline in the context of personality has now become a toxic term that is hindering progress in research and treatment.

The only accurate aspect of borderline is its title, a word that correctly signifies its complete lack of specificity. It emerged over 60 years ago to describe patients on the border between neurosis and psychosis who might be amenable to treatment with psychoanalysis. Not surprisingly, its diagnostic criteria are not longstanding personality dispositions, but oscillating symptoms and behaviour.^2^ The triad of unstable mood, erratic relationships and disturbed behaviour may be readily identifiable but that does not make it a personality disorder; chronic sleep disturbance creates the same symptoms. A constant and undisputed diagnostic aspect of true personality disturbance is the presence of traits, characteristics reflecting individual function, which are generally stable over time and, when disturbance becomes disorder, are maladaptive. The widely gyrating features of emotional instability do not belong in this paradigm.

The diagnosis of borderline or emotionally unstable personality disorder in the major DSM-III revision of classification in 1980 was only introduced as a grubby compromise to satisfy psychoanalysts who were unhappy with an atheoretical classification system. Revisions of ICD-10 and DSM-IV have highlighted the failures of previous labels. Both classification committees favoured a dimensional representation of personality pathology consistent with current evidence. Such a model implies that the central features of abnormal personality should be present, albeit to a lesser degree, across the range of personality disturbance. Both recently accepted classifications of personality disorder, ICD-11 (World Health Organization) and the American DSM-5 Alternative Model of Personality Disorder have trait domains that link well with the commonly described Big Five domains of normal personality.^3^ All attempts to find a borderline factor have failed.^4^ If borderline was a true personality disorder, it would not be outside this system. Many clinicians and patients attune to the diagnostic descriptions of borderline features, as their features are easy to detect and very common from adolescence onwards and the diagnosis seems to give a reassuring degree of certainty to otherwise intangible complex symptoms and behaviour.^5–6^ But any positive aspects are overcome by its contradictions and the confusion created by overlap. It is a mushy blancmange diagnosis that simply embraces too much pathology to be of any real value. Although borderline symptoms make sense as a syndrome examined in isolation, they disappear into a general factor when modelled alongside other personality disorders.^7^ The overlap of borderline features with almost every other psychiatric disorder, particularly ADHD, bipolar disorder and other mood disorders,^8–9^ also muddies the diagnostic waters. Both ICD-10 and ICD-11 diagnostic work groups rejected borderline and emotional instability in their classifications but they were forced in by powerful lobbies. It is not just Big Pharma that can influence diagnostic practice.

Because the criteria for diagnosis are so overinclusive – including anxiety and depressive moods, identity disturbance and psychotic features – even if there is no personality pathology, a borderline diagnosis could be made. Making a borderline diagnosis obscures rather than illuminates pathology.

One strong claim in favour of the diagnosis of borderline personality disorder is that it is linked to specific treatments. But the evidence of their efficacy has been overstated. When the patina of language such as dialectic, mentalisation, schema formation and transference is stripped away, the treatments offered are exactly the same as those offered for general psychological distress and dysfunction, now given an unnecessarily new title, structured clinical management. The methods used to reduce distress are transdiagnostic and apply to all patients. No medications have been found to be of any consistent benefit in the treatment of borderline personality disorder, and the two largest and best designed studies using olanzapine and lamotrigine were unequivocally negative.^10,11^ Despite this, almost all patients with the disorder appear to receive not just one, but many psychotropic drugs for this condition and several US guidelines continue to recommend drug combinations for the condition. A just published Cochrane review concluded that ‘no pharmacological therapy seems effective in specifically treating BPD pathology’.^12^

The indiscriminate use of borderline in multiple contexts is a major source of stigma. Those with emotional instability, a syndrome that undeniably exists but is best thought of as a mood disorder, are combative and often eloquent in seeking care, and employ what were once called ‘immature defences’ such as splitting and projection. Put in simpler form, they distract and annoy the clinician.^1^ There are many other reasons patients challenge their doctors, but in the current climate, this behaviour – whether expressed in accident and emergency departments, general practitioners’ surgeries or psychiatric settings – leads to eye-rolling, nods and winks to colleagues and the whispered comment ‘another borderline’ that foreshadows inappropriate and unsympathetic intervention. Health professionals are the worst offenders in promoting stigma and the consequent angry reactions it provokes. As a consequence, the patients so identified are seen as more difficult to manage even when their behaviour is no worse than other patients who are not labelled with borderline pathology.^13^ It also makes it more difficult for these patients to have other psychiatric disorders recognised such as depression, anxiety and ADHD. Patients frequently complain that when they mention these other problems, they get responses such as ‘this is all part of your emotional instability diagnosis’ or ‘once we sort out the borderline problem these will disappear’. It is almost as though the mere hint of borderline pathology devalues all other symptoms, not just psychiatric but also medical, on the grounds that they are exaggerated and distorted and can be conveniently disregarded and attention given to more needy patients.

Increasingly, mention of ‘emotional instability' in correspondence about a patient will be used to exclude the patient from a range of mental health services on spurious grounds of inappropriate behaviour or diagnostic mismatch. This only serves to increase the sense of alienation that many already feel and the sad fact is that now any mention of emotional instability is a major source of refusal to treat by many parts of the psychiatric service. This reinforces the view that the diagnosis of borderline is being used increasingly as one of exclusion; this only serves to increase the sense of alienation and anger by sufferers. As only a tiny proportion of potential referrals can be treated by specialist services,^15^ accentuating these feelings as rejection from those services will become the norm.

The new ICD-11 personality disorder classification takes a broader assessment far beyond that of ticking off a set of borderline operational criteria. The new dimensional classification – all of us are on a personality spectrum – leads to a more nuanced assessment of a patient’s psychopathology that extends far beyond borderline pathology. Clinicians begin by assessing the level of severity of personality dysfunction into four groups of severity that lead to the diagnosis and this is then qualified by the presence of one of five domains similar to the Big Five of normal personality variation. A ‘borderline pattern specifier’ has been added for those who feel they cannot yet dispense with the syndrome even though all the relevant pathology can be captured in ICD-11 without requiring its use.^15^ Most patients present acutely in emergency departments after self-harm, and similar crises are likely to have moderate personality disorder, as this is characterised by multiple areas of functioning and relationships, often associated with harm to self or others with significant impairment in most areas of life.

A more sophisticated formulation might lead to a different range of interventions rather than a standard protocol-driven treatment given to all patients^16^ with, not surprisingly, similar outcomes. For example, patients with less severe borderline pathology, largely involving negative affectivity, might be able to benefit from less structured and intense therapy, possibly in groups. Those with evidence of disinhibition, and particularly dissociality, might benefit from individual treatment which is highly structured and transparent with clear boundaries. Those with identity disturbance and dissociation may need more trauma-focused treatment. These statements are obviously speculative but continuing to lump all patients together with a borderline diagnosis does not allow the model to progress to tailored individual treatments.

The diagnosis of borderline, of emotionally unstable, personality disorder is widely and inappropriately used, informs little, creates confusion and uncertainty, and generates tremendous stigma. It has no basis in the scientific study of personality and is used indiscriminately to describe myriad negative interactions in human relationships that have cause far beyond personality function, extending from simple disagreement to total functional breakdown. Because of its profligate usage and scientific inaccuracy, the management and specific treatment of this group of conditions is severely compromised and has become a major bar to understanding. Borderline no longer has a place in clinical practice.

## Declarations

### Guarantor

RM and PT.

### Provenance

Not commissioned; editorial review.
